# Long-term surgical anaesthesia with isoflurane in human habituated Nile Crocodiles

**DOI:** 10.4102/jsava.v88i0.1451

**Published:** 2017-02-24

**Authors:** George F. Stegmann, Catherine J.A. Williams, Craig Franklin, Tobias Wang, Michael Axelsson

**Affiliations:** 1 Department of Companion Animal Clinical Studies, University of Pretoria, South Africa; 2 Department of Bioscience, Aarhus University, Denmark; 3 School of Biological Sciences, University of Queensland, Australia; 4 Department of Biological and Environmental Sciences, University of Gothenburg, Sweden

## Abstract

A suitable long-term anaesthetic technique was required for implantation of physiological sensors and telemetric devices in sub-adult Nile crocodiles (*Crocodylus niloticus*) to allow the collection of physiological data. Five Nile crocodiles with a median body mass of 24 kg were used. After manual capture, they were blindfolded and 0.2 mL (1 mg/mL) medetomidine was administered intramuscularly in four of the animals which had an estimated body mass between 20 kg and 30 kg. One crocodile with an estimated body mass of 50 kg received 0.5 mL. For induction, 5 mL propofol (10 mg/mL) was injected intravenously into the occipital sinus. Additional doses were given when required to ensure adequate anaesthesia. Anaesthesia was maintained with 1.5% isoflurane. Ventilation was controlled. Local anaesthesia was administered for surgical incision and external placement of the radio transmitter. Medetomidine was antagonised with atipamezole at the end of surgery. Median heart rate during surgery was 22 beats/min, at extubation 32 beats per min and 30 beats per min the following day at the same body temperature as under anaesthesia. Median body temperature of the animals increased from 27.3 °C to 27.9 °C during anaesthesia, as room temperature increased from 24.5 °C to 29.0 °C during surgery. Anaesthesia was successfully induced with intramuscular medetomidine and intravenous propofol and was maintained with isoflurane for the placement of telemetric implants. Intraoperative analgesia was supplemented with lidocaine infiltration. Perioperative physiological parameters remained stable and within acceptable clinical limits. Multiple factors appear to influence these variables during the recovery period, including residual anaesthetic effects, environmental temperature and physical activity.

## Introduction

Long-term anaesthesia was required to facilitate the implantation of physiological sensors and telemetric equipment in Nile crocodiles (*Crocodylus niloticus*) for the measurement of physiological variables. Reports in the literature focus on the capture and immobilisation of crocodiles, and anaesthetic techniques for long-term surgical anaesthesia in crocodiles have not been described.

Various injectable anaesthetic drugs and peripheral muscle relaxants have been used for chemical immobilisation and anaesthesia of crocodilians. These included drugs such as atracurium (Clyde, Cardeilhac & Jacobson 1994) and gallamine (Fleming 1996), etorphine (Hinsch & Gandal 1969), tiletamine and zolazepam (Clyde et al. 1994; Jacobson 1984) as well as ketamine and medetomidine (Dennis & Heard 2002; Heaton-Jones, Ko & Heaton-Jones 2002; Olsson & Phalen 2012a, 2012b, 2013) and propofol (Divers 1996). Isoflurane is commonly used for the maintenance of anaesthesia in crocodiles (Heard & Stetter 2007; Mosley, Dyson & Smith 2004).

In this report, the methodology and physiological responses are described during prolonged balanced anaesthesia and recovery in sub-adult Nile crocodiles.

## Materials and methods

Five sub-adult Nile crocodiles (*C. niloticus*) of unknown age with a median (range) body weight of 24 kg (21, 47) were obtained for this investigation from a commercial crocodile breeding farm. The animals were habituated to human presence and were moved to a wildlife quarantine facility near Hammanskraal, North West Province, South Africa a month before the investigation.

During the perioperative period, the animals were kept in a fenced camp that included a pool for the animals to access *ad lib*. The crocodiles were fasted for 3 days before the project started. The weight of the animals was estimated before anaesthesia and then accurately determined with an electronic scale after induction of anaesthesia. For physical immobilisation, a team member approached from the tail end and placed a blindfold over the head. Four team members followed to manually immobilise the head, thorax, tail and legs. The jaws were immediately taped closed caudal to the nostrils with PVC electrical insulation tape. Four of the animals were captured beside the pool in this manner. The fifth crocodile was captured in the pool with a catch pole. The noose was placed around the neck for removal from the pool. At the time of physical immobilisation, medetomidine (Domitor 1 mg/mL, Zoetis, Sandton) was administered intramuscularly in the thoracic limb muscles close to the base of the neck. A dose of 0.2 mL was administered to four of the animals whose estimated weight ranged between 20 kg and 30 kg and 0.5 mL was administered to the fifth animal whose estimated weight was 50 kg. Anaesthesia was induced with the intravenous injection of propofol (Propofol Fresenius 10 mg/mL, Fresenius Kabi, Midrand) into the occipital sinus (Myburgh et al. 2014). A 30 mm, 23 G hypodermic needle attached to a 5 mL syringe filled with propofol was used for venipuncture. The needle was introduced in the dorsal midline through the skin approximately 2 mm caudal to the cranial scute aimed in a caudal direction at an angle of 85° to the skin surface. The needle was advanced to a depth of approximately 20 mm. During introduction of the needle, negative pressure was applied by slightly pulling back on the syringe plunger. When the needle entered the occipital sinus, blood was observed at the hub of the needle at the base of the syringe. Five millilitre of propofol was injected as a single bolus over a period of 60 s and a period of 2–3 min was allowed for onset of anaesthesia. If the anaesthetic depth was inadequate to allow transport, further boluses of 2.5 mL propofol were administered to effect. Once adequately anaesthetised, the needle was removed from the sinus and the crocodile placed on a canvas stretcher to be transported on the back of an open pickup truck for a distance of 500 m to the surgical facility.

At the facility, the trachea was intubated for maintenance of anaesthesia with isoflurane (Isofor, Safeline, Weltevreden Park) and oxygen delivered by a circle rebreathing circuit with soda lime for CO_2_ absorption. For intubation, the electrical tape was removed from the jaws and a 200 mm long, 25 mm diameter plastic water pipe was unilaterally placed as a gag to keep the jaws open. The palatal flap was displaced from the soft palate to visualise the larynx. After intubation, a 5 cm long PVC pipe was lengthwise taped in at the tip of the jaws through which the tracheal tube was stabilised. During inhalation anaesthesia, the pelvic limbs were taped to the body wall with duct tape. For maintenance of anaesthesia, the vaporiser (Fortec Mk III) was initially set at 4% isoflurane with the fresh gas flow rate at 2 L/min. Manual positive pressure ventilation (PPV) was applied at 6 breaths per min until the pedal reflex was absent. Tidal volume was adjusted until the start of body wall expansion was observed. Then, the vaporiser setting was stepwise reduced to 1.5% with 10 min intervals and the fresh gas flow rate reduced to 1 L/min. Manual PPV was applied at 1 breath per min during surgery. During closure of the sternum, the fresh gas flow rate was increased to 3 L/min, the isoflurane concentration reduced to 1% and finally switched off during surgical closure of the skin. The breathing circuit was flushed with oxygen. Then, PPV was reduced to 1 breath per 5 min and the animal was observed for return of spontaneous ventilation. At the end of surgery, the tracheal tube was disconnected from the anaesthetic machine. Positive pressure ventilation was applied with an Ambu-bag until extubation. The animal was returned to the recovery pen. The trachea was extubated after return of spontaneous ventilation. For recovery, the animals were housed singly in small pens for 2 days before access to the pool was allowed.

Enrofloxacin (Baytril 5%, Bayer AH, Isando) was injected intramuscularly at the start of surgery at a dose of 5 mg/kg body mass. At completion of surgery, tulathromycin (Draxxin 100 mg/mL, Zoetis, Sandton) at 2.5 mg/kg and flunixin meglumine (Finydine 50 mg/mL, Intervet SA, Isando) at 0.2 mg/kg were intramuscularly administered. A line block with 5 ml lidocaine (Lignocaine 2%, Bayer AH, Isando) was infiltrated along the ventral midline before surgical incision. At the end of surgery, 5 mL lidocaine was infiltrated below the four dorsal nuchal scutes to attach the external time-depth recorders with an inbuilt very high frequency (VHF) transmitter.

Anaesthetic depth was monitored by observing eyelid closure, absence of the palpebral reflex, muscle relaxation in the limbs and tail. During surgical anaesthesia, absence of the pelvic limb withdrawal (pedal) reflex when applying manual finger pressure for 5 s over the second digit was maintained. A multi-parameter monitor (Advisor Vital Signs Monitor, Smiths Medical, OH, USA) was used to monitor end-tidal CO_2_ (ETCO_2_) partial pressure, oxyhaemoglobin saturation (pulse oximetry) and body temperature. The side-stream capnometer sampling line was attached to the tracheal tube, the pulse oximeter reflectance probe taped to the hard palate and the temperature probe placed in the cloaca. Duration of inhalation anaesthesia was measured from the time the animal was connected to the inhalation anaesthetic machine until it was disconnected from the machine.

For the telemetric implants (Endosmatic Systems Inc., Davis, CA, USA), the ventral midline over the sternum was surgically prepared and incised. The left and right aortic arches were dissected clean and the pericardium opened for implantation of various sensors of the biotelemetry system. Two Doppler flow probes were placed on the right and left aortae, respectively, and two 3F pressure catheters were inserted through the wall of the outflow tract into the pulmonary and right aortic outflow region and secured using a purse-string suture and an additional suture at the base of the outflow tract (Millar Instruments, Houston, TX, USA). Two electrocardiogram wires were also attached to the pericardium. The body temperature sensor was imbedded in the implant case. The implant was connected to a data logger and battery pack and was placed in the abdomen. A VHF transmitter that incorporated the time-depth recorder and environmental temperature recorder (Titley Electronics, Columbia, MO, USA) was externally sutured to the nuchal scutes. The implanted equipment allowed mid-anaesthetic, extubation and recovery values for heart rate (HR), systemic mean arterial blood pressure (MAP) and respiratory rate to be monitored. Data recovered from the data loggers are presented here for the first 24 hours covering anaesthesia, extubation and recovery. The medetomidine was reversed with intramuscular atipamezole (Antisedan 5 mg/mL, Zoetis, Sandton) at 50 µg/kg at completion of surgery. In Crocodile 2, atipamezole was given 40 min after the start of surgery because of depression of cardiac force of contraction, as observed intraoperatively by the surgeons. The University of Pretoria’s Research and Animal Ethics Committees approved the protocol (V072/13).

## Results

**FIGURE 1:**
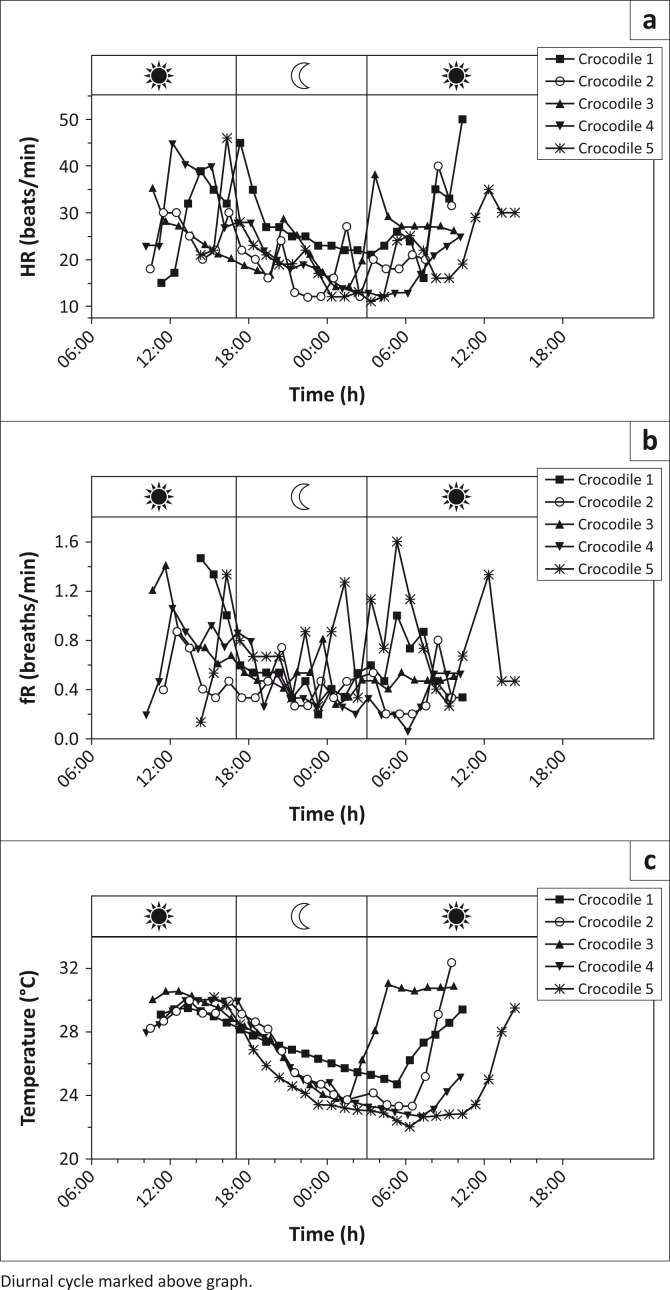
Graphs of (a) heart rate (beats per min), (b) respiratory frequency (breaths per min) and (c) internal body temperature (°C) of the five crocodiles from anaesthesia to recovery over 24 hours (UTC).

**FIGURE 2:**
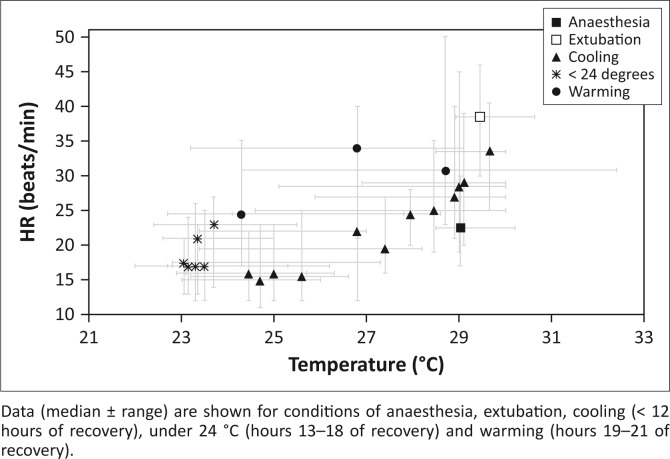
Plot of the relationship between heart rate (beats per min) and internal body temperature (°C) for crocodiles 1, 2, 4 and 5 from their anaesthesia through recovery, showing evidence of heart rate hysteresis dependent on cooling or warming state, and separation of anaesthesia and extubation conditions from the trend for recovery.

The median (range) dose for medetomidine was 8.3 µg/kg (6.3, 10.6), and for propofol it was 2.1 mg/kg (1.6, 3.8). HR, respiration rate (fR) and body temperature from the individual animals were plotted over time in Figure 1, with the first point on each line representing surgical plane of anaesthesia. These graphs are shown relative to the diurnal cycle. Missing data for respiratory rate reflect where a respiratory pattern could not be derived from the pulmonary pressure data recovered from the loggers. In Figure 2, HR was plotted against body temperature (median, range) for the first 24 hours of data, including anaesthesia 60 min before extubation, at extubation and hourly during recovery.

During surgical preparation, the HR from only three of the five animals was obtained using the pulse oximeter and was 22 beats per min, 20 beats per min and 33 beats per min, respectively. During surgical anaesthesia (*n* = 5) 1 h prior to extubation, the median (range) HR was 21 (16, 33 from implant data) beats per min. At the end of anaesthesia, the median HR was 21 (15, 31), at extubation: 32 (27, 46) and 24 hours after recovery: 30 (23, 50) beats per min. The median (range) for ETCO_2_ during surgery was 25 (18, 30), 13 (8, 30), 25 (20, 35), 24 (20, 25) and 25 (20, 30) mmHg for Crocodiles 1–5, respectively.

MAP (*n* = 2) during surgery was 34 and 20 mmHg that increased to 47 mmHg and 64 mmHg, respectively, at extubation. The MAP for hourly measurements during the first 24 hours after extubation was 45 mmHg and 52 mmHg, respectively. Median (range) resting respiratory frequency 24 hours after anaesthesia was 0.46 breaths per min (0.33, 0.53). Median body temperature of the animals increased from 27.3 °C to 27.9 °C during anaesthesia, and median room temperature increased from 24.5 °C to 29.0 °C. Median (range) for duration of inhalation anaesthesia was 145 min (123, 195). Parameters varied over the first 24 hours from recovery with degree of sedation, activity and with diurnal pattern dictating environmental temperature.

## Discussion

The purpose of anaesthesia was to facilitate implantation of data loggers to collect continuous physiological data over an extended period from crocodiles bred in captivity that had free access to a pond. It also provided a unique opportunity for the collection of physiological data associated with anaesthesia during the 24 hours perianaesthetic period. For this investigation, anaesthesia was induced with intravenous propofol and medetomidine co-administered intramuscularly at induction. Then, anaesthesia was maintained with isoflurane during inhalation anaesthesia. Analgesia was provided with medetomidine and lidocaine local infiltration. No intraoperative responses to surgery were observed, such as reflex movement or increased heart or ventilation rates. Postoperatively, flunixin was administered before the animals were returned to the pens to recover from anaesthesia. Postoperatively, the behaviour of the animals was similar to the adaptive period before surgery.

Medetomidine is a α_2_-adrenergic agonist that results in sedation, analgesia, muscle relaxation and an anaesthetic sparing effect (Cullen 1996). Dose values reported previously for medetomidine in crocodiles were intended for immobilisation, capture and anaesthesia (Heaton-Jones et al. 2002; Olsson & Phalen 2012a, 2012b, 2013). In this investigation, a low-dose medetomidine of 1 µg/kg was arbitrarily selected as it only served to prolong anaesthesia after propofol induction, minimise risk of recovery from anaesthesia during transport to the surgical facility and give intraoperative analgesia. Propofol induction should preferentially be performed after the full onset of medetomidine. In this investigation, propofol induction was performed directly after intramuscular medetomidine as intravenous access for injection was only available for a short period during induction of anaesthesia directly after capture.

The dose values reported previously for propofol for induction of anaesthesia ranged from 10 mg/kg to 15 mg/kg (Heard & Stetter 2007). However, Fleming (2014) was of the opinion that doses as low as 5 mg/kg may be suitable for tracheal intubation. In this instance, the median dose required to intubate the animals was 2.1 mg/kg. The possibility that medetomidine had already influenced the propofol dose may not be ignored, although propofol was administered within 5 min of intramuscular medetomidine. Intubation was only performed after arrival at the surgical facility. Human habitation also limited capture stress and could contribute to a reduction in the induction dose.

Values for HR, fR and body temperature observed in this investigation were similar to values reported for juvenile alligators anaesthetised with medetomidine and ketamine (Heaton-Jones et al. 2002). The pulse oximeter was used to obtain values for HR in the preoperative period only. After implantation, the values for HR and MAP were obtained from the data loggers. Mean arterial pressure recorded from the aortic arch from two animals in this investigation was 20 mmHg and 34 mmHg, respectively, and was similar to a MAP of approximately 21 mmHg recorded previously from the right aortic arch in a caiman (*Caiman crocodilus*) induced with methohexital and maintained with halothane (Axelsson, Holm & Nilsson 1989). Haemoglobin saturation values were considered inaccurate because of differences in haemoglobin structure between domestic animals (mammals) and reptiles (Mosley et al. 2004). The observed saturation values displayed by the pulse oximeter for all the five animals were approximately 50% despite the animals breathing pure oxygen. This could also be because of shunting while under anaesthesia and the relatively poor penetration of the equipment in reptilian soft tissue. The period when it was possible to obtain a pulse rate was relatively short and limited to the period after induction of anaesthesia during surgical preparation. It is assumed that peripheral vasoconstriction induced by medetomidine (Cullen 1996) was responsible for the short period when it was possible to obtain a pletysmographic waveform and HR from the pulse oximeter. HRs during anaesthesia tended to be lower in comparison with other data collected at the same temperature during recovery (Figures 1 and 2).

The crocodile is poikilothermic and it is expected that environmental temperature will influence the rate of recovery from anaesthesia. In this instance, the majority of animals were anaesthetised in the morning and anaesthesia terminated around midday when day environmental temperatures were at their highest. A marked increase in body temperature was observed in the animals induced in the early morning (Crocodiles 1–4) when environmental temperatures were far below midday temperatures, whereas Crocodile 5 was induced around 13:00. Cooling over time was more uniform than warming between crocodiles (Figure 1), with crocodiles starting to warm at different points in the day following surgery and at different rates. Maintenance of a species’ preferred body temperature should allow optimal anaesthetic recovery. Medetomidine may inactivate body temperature regulation in mammals, but the influence of α_2_-adrenergic agonists on body temperature regulation in crocodiles has not been previously reported, although medetomidine has been recently investigated as a sedation agent for captive and free-living crocodilians (*Crocodylus porosus* and *Crocodylus johnstoni*), and its effects at different initial body temperature conditions reported (Olsson & Phalen 2012a, 2012b). In our study, lower doses were used, as medetomidine was not the sole immobilisation agent, but also to limit the reduction in HR and blood pressure seen when used as the sole agent, and to allow rapid restoration of normal physiological function. In our investigation, the antagonist atipamezole was administered to all animals before or at the end of anaesthesia to optimise recovery.

The consideration of the relationship between HR and body temperature in the first 24 hours after anaesthesia is interesting not only in terms of the anaesthetic, but also given crocodilian thermoregulatory mechanisms. These include HR hysteresis, that is, the finding that the HR is higher at the same temperature during warming than in a cooling phase. This functionally allows reptiles to extend the time period over a day when they are within their preferred thermal range (Axelsson et al. 1989; Franklin 2003; Seebacher & Franklin 2005). Some evidence of HR hysteresis in the warming and cooling phases of recovery was observed in this study (Figure 2). The resumption of a normal hysteresis cycle may reflect early return to normal physiology; however, it may also be influenced by behaviour, and anaesthetic and analgesic drugs used. Crocodile 3, which was the largest specimen, was noted on video recordings to have undergone a period of strenuous activity 10 h after implantation (while body temperature was falling), which is reflected in this animal’s HR (Figure 1). This crocodile also showed an increase in body temperature prior to sunrise the day after surgery, which we propose may be because of increased muscle activity involved in attempted territorial dominance. Crocodiles have a large anaerobic exercise capacity and high tolerance for lactate. Crocodiles (*C. porosus*) of this weight (50 kg) have been published to have a total power output from exercise similar to that of the aerobic power output of a similarly sized mammal, with consequent heat production (Seymour 2013). Large body size may then allow this elevated temperature to be maintained through inertial homeothermy. This crocodile also had a higher body temperature during morning surgery than the smaller individuals (first data point Figure 1), again potential evidence for more efficient inertial homeothermy in larger individuals (Seymour 2013). Crocodile 3 was excluded from the analysis for HR hysteresis as the warming phase for this animal was distinct (Figure 1), as it was not apparently environmentally initiated.

A confounding factor in the relationship between temperature and HR during the first 24 hours of recovery may be the waning effect of the anaesthetic drugs, as the warming period is necessarily later than the cooling, and therefore may be less affected by drugs given at the time of surgery. The anaesthetic may affect the ability of the crocodile to physiologically regulate its cardiac parameters as it would normally. Concurrent influences of temperature, recovery from isoflurane, the use of flunixin and medetomidine make definitive statements problematic.

The crocodiles in this study received an injection of flunixin meglumine, a potent non-steroidal anti-inflammatory, at the end of surgery to provide postoperative analgesia. The presence of HR hysteresis in a crocodilian treated with a prostaglandin antagonist is in line with previous studies; prostaglandin antagonists such as non-steroidal drugs have been found to abolish the HR hysteresis in *Pogona vitticeps*, but not in crocodilians (Seebacher & Franklin 2003).

Given the findings that crocodilians may rely mainly on central autonomic mechanisms for thermoregulation (Seebacher & Franklin 2003, 2004), medetomidine may be expected to change their ability to thermoregulate as, after an initial increase, it causes depressed blood pressure and HR in those animals where it has been measured (Jalanka & Roeken 1990; Sinclair 2003; Sleeman & Gaynor 2000). The antagonist for medetomidine, atipamezole, did not fully reverse the cardiopulmonary effects of medetomidine in a tortoise (Sleeman & Gaynor 2000). This is interesting considering the presence of a degree of HR hysteresis during the first 24 hours after administration of medetomidine and atipamezole in these crocodiles. The crocodiles were able to maintain hysteresis in the presence of drugs, which would be expected to influence blood pressure and HR. The influence may be limited by drug dose, as the low-dose medetomidine was calculated on estimated weight.

Medetomidine was antagonised with atipamezole at five times the medetomidine dose based on body mass. Reported doses for antagonism were five times the dose of medetomidine based on body surface area (Heaton-Jones et al. 2002) or only on body mass (Fleming 2014). The administration of atipamezole may have contributed to the rise of HR from anaesthetic values to extubation values; however, there is a rise even in Crocodile 2 where medetomidine was reversed earlier. Therefore, increased levels of sympathetic stimulation because of decrease in anaesthetic depth and stimulus from the tracheal tube and surroundings are most likely to contribute towards this rise. An exception was Crocodile 3, which showed an increased HR during anaesthesia in comparison with extubation.

Manual PPV was applied at 1 breath per min as increasing the rate resulted in clinically significant decreases in ETCO_2_ values that raised concerns that this might further contribute to delays in return of spontaneous ventilation at the end of surgery. After terminating isoflurane administration, the ventilation rate was reduced to allow for the onset of spontaneous ventilation. The administration of isoflurane was also ended during skin suturing to allow for anaesthetic gas elimination and decreased anaesthetic depth before decreasing the ventilation rate.

## Conclusion

For induction of anaesthesia, medetomidine was co-administered intramuscularly with the intravenous injection of propofol to facilitate tracheal intubation. The median dose for medetomidine was 8.3 µg/kg body mass and for propofol it was 2.1 mg/kg. Anaesthesia was maintained with 1.5% isoflurane. Ventilation was controlled at a rate of 1 breath per min to maintain a median ETCO_2_ partial pressure of 25 mmHg. HR remained stable during surgery at a median rate of 22 beats per min, only to increase at extubation to a median of 32 beats per min when spontaneous ventilation returned at a light plane of anaesthesia and to resume a pattern of temperature-dependent hysteresis. Median body temperature increased from 27.3 °C to 27.9 °C during anaesthesia, whereas median room temperature increased from 24.5 °C to 29.0 °C. Multiple factors appear to influence physiological variables during the recovery period including residual anaesthetic effects, environmental temperature and physical activity.
